# Electrospun Polymer Nanofibers Decorated with Noble Metal Nanoparticles for Chemical Sensing

**DOI:** 10.1186/s11671-017-2216-4

**Published:** 2017-07-11

**Authors:** Chen Chen, Yongan Tang, Branislav Vlahovic, Fei Yan

**Affiliations:** 1 Department of Chemistry and Biochemistry, North Carolina Central University, Durham, North Carolina 27707 USA; 2 Department of Mathematics and Physics, North Carolina Central University, Durham, North Carolina 27707 USA

**Keywords:** Electrospinning, Polymer nanofibers, Noble metal nanoparticles, Sensing

## Abstract

The integration of different noble metal nanostructures, which exhibit desirable plasmonic and/or electrocatalytic properties, with electrospun polymer nanofibers, which display unique mechanical and thermodynamic properties, yields novel hybrid nanoscale systems of synergistic properties and functions. This review summarizes recent advances on how to incorporate noble metal nanoparticles into electrospun polymer nanofibers and illustrates how such integration paves the way towards chemical sensing applications with improved sensitivity, stability, flexibility, compatibility, and selectivity. It is expected that further development of this field will eventually make a wide impact on many areas of research.

## Review

### Background

The rapid development of nanoscience and nanotechnology has led to a wide variety of practical applications, including air filtration, wound dressings, drug delivery, detection, energy production, and food packaging [1–10]. Nanomaterials often have physical and chemical properties that are very different from the same materials at larger scales. Many different strategies have been developed for the synthesis and construction of nanostructured materials [11–13]. Based on dimensionality, nanomaterials may be classified into four categories: zero-dimensional (0D), one-dimensional (1D), two-dimensional (2D), and three-dimensional (3D). 1D nanomaterials such as nanowires, nanorods, and nanotubes have been widely investigated in the last few decades. Among the aforementioned materials, 1D nanofibers have attracted tremendous attention due to their unique structural and physical properties such as small diameters, large surface area per unit mass, small pore size, and flexibility in surface functionalities [14, 15]. There are many processing techniques that have been utilized to produce 1D nanofibers such as template synthesis [16], self-assembly [17], and electrospinning [18, 19]. Among these methods, electrospinning appears to be the most versatile and simplest one for preparing nanofibers [15]. It is notable that by adjusting the parameters of the polymer solution or the electrospinning setup, most of the known polymers such as polyacrylonitrile (PAN) [20, 21], polyvinylidene fluoride (PVdF) [18], and polyvinylalcohol (PVA) [22] can be successfully electrospun into ultrafine fibers. Therefore, due to the significant simplicity and versatility of electrospinning, electrospun polymer nanofibers have garnered substantial attention in recent years, particularly in the field of chemical sensors.

The field of plasmonics that deals with light-matter interactions between adsorbed molecules and noble metal structures at nanoscale dimensions has recently emerged as a rapidly growing area of interest, as evidenced by the explosive growth in various fields including surface-enhanced Raman scattering (SERS) [23–25], surface-enhanced infrared absorption spectroscopy [26, 27], surface-enhanced fluorescence spectroscopy [28–30], surface plasmon resonance spectroscopy [31–34], and plasmonic colorimetry [35]. The fascinating optical properties of plasmonic nanostructures are dominated by collective oscillations of the conduction band electrons in the noble metal (e.g., Au, Ag, and Pt) nanostructures known as surface plasmons. The quest for simple methods to fabricate reproducible plasmonic nanostructures has spurred much interest in a variety of scientific disciplines; however, it has remained a big challenge to hierarchically assemble individual noble metal nanostructures with desirable long-range order at predefined sites on a substrate. Templated synthesis and assembly of nanoscale plasmonic building blocks to form rationally designed architectures have emerged as an overarching strategy for addressing this challenge [36–38]. Electrospun polymer nanofibers have been shown to be one of the most promising templates to pack noble metal nanostructures with great precision. The controlled incorporation of noble metal nanostructures with desired plasmonic properties into electrospun polymer nanofibers paves the way towards sensing applications with improved sensitivity, stability, flexibility, compatibility, and selectivity.

This review highlights recent advances in integrating electrospun polymer nanofibers with noble metal nanoparticles and their applications for chemical sensing. We summarize the following: (1) the basic setup and process parameters for electrospinning, (2) different strategies for the synthesis of Au or Ag nanostructures, (3) preparation of electrospun polymer nanofibers decorated with Au or Ag nanoparticles, and (4) examples of chemical sensing applications of electrospun polymer nanofibers decorated with Au or Ag nanoparticles.

## Electrospinning: Basic Set-up and Process Parameters

**Fig. 1:**
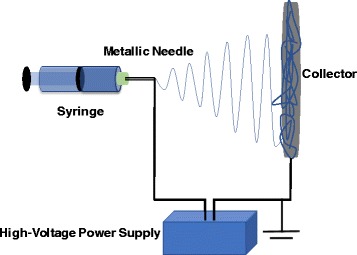
The basic laboratory setup for electrospinning

The electrospinning system generally consists of four main parts: a direct current power supply with high voltage, a syringe that contains polymer solution, a metallic needle with a blunt tip, and a grounded conductive collector, as shown in Fig. 1. During the electrospinning process, the polymer solution in the syringe will be pumped out through the metallic needle tip at a specific rate. A high voltage is applied to create charges on the surfaces of the polymer droplet forming a Taylor cone, and when the repulsive electrostatic force is sufficiently strong to overcome the surface tension of the polymer droplet, the polymer droplet will be elongated into a conical shape [39]. Subsequently, the polymer jets will undergo an elongation process, during which the polymer will be stretched and the polymer solution solvent will evaporate, leaving the long and thin polymer nanofibers collected on the grounded conductive collector.

One of the great advantages of electrospinning is that by changing the parameters during the electrospinning process, the morphology of the electrospun nanofibers can be easily controlled. These parameters include polymer concentration, solution viscosity, solution conductivity, flow rate, applied voltage, the working distance between the collector and tip of the needle, and air humidity [12]. Polymer concentration is an important parameter as it determines the morphology of the electrospun nanofibers, because the surface tension can be dominant as the polymer concentration decreases, which will lead to polymer bead formation [39]. In addition, solution viscosity is another critical parameter, which determines if the polymer can be elestrospun into nanofibers or not. The solution viscosity is highly dependent on the polymer concentration and the molecular weight of the polymer used for the electrospinning. In principle, a polymer with higher molecular weight has, on average, longer molecular chains, and it will form more entanglements leading to a higher viscosity of the polymer solution. Therefore, for a solution made with high molecular weight polymers, even though the polymer concentration is low, it still can produce a uniform jet due to a sufficient level of solution viscosity. Conversely, if the molecular weight is too low, an appropriate polymer solution viscosity cannot be guaranteed even with a high polymer concentration and the polymer tends to form a bead structure on the collector [40]. Comparatively, the processing conditions such as applied voltage and flow rate also play a significant role in nanofiber formation during electrospinning. For applied voltage, it has been proven that varied applied voltage will not change the nanofiber morphology dramatically. According to the past work, both larger and smaller fiber diameters can be obtained when a higher voltage is applied [40].

## Synthesis and Assembly of Au Or Ag Nanostructures

During the last few decades, great advances have been made in the synthesis of Ag and Au nanostructures with different sizes and shapes. It is worth noting that different nanostructures can give rise to significantly different optical, electronic, magnetic, or chemical properties, which may be suitable for different applications. Generally, based on the different mechanisms, the reductive approaches to Au or Ag nanostructures can be approximately classified into chemical and physical methods. Typically, the way to obtain Au or Ag nanostructures is to mix Au or Ag precursors with a reducing agent and/or a colloidal stabilizer, and nanostructured Au or Ag with different sizes and shapes can be generated under specific conditions. AgNO_3_ and HAuCl_4_ are the most commonly used precursors for Ag and Au nanostructure synthesis, and various reducing agents such as sodium borohydride, alcohols, sodium citrate, and poly(vinyl pyrrolidone) (PVP) can reduce Ag/Au ions into Ag/Au atoms with exceptional control over their sizes and shapes. It has been proved that the plasmon resonance frequencies of the Au or Ag nanoparticles depend on their sizes. For example, Xia and coworkers have synthesized Ag nanocubes ranging from 60 to 100 nm and compared their SERS with respect to both size and shape (sharp vs. truncated) [39]. It demonstrates that larger particles (90 and 100 nm) were found to have higher SERS efficiencies (90 and 100 nm), which is primarily attributed to the overlap between the laser source and plasmon resonance band. Additionally, particles with shaper corners also gave more intense SERS signals than their truncated counterparts.

### Synthesis of Au Nanostructures

Based on Turkevich’s research in 1951, HAuCl_4_ could be reduced in a water solution in the presence of citrate, which has been one of the most commonly used methods for Au nanoparticle synthesis [41]. By changing the amount of citrate, the mean size of the Au nanoparticles can be easily manipulated and citrate plays a role as a nucleating agent and a growth agent at the same time [41]. It has been proven that the citrate reduction method can produce relatively narrow size distributions of the Au nanoparticles. Subsequent studies demonstrated that the mechanism of the control on different Au nanoparticle sizes as a function of the amount of citrate is intimately related to the pH values, because different pH values will determine the formation process of the Au nanoparticles [42].

**Fig. 2:**
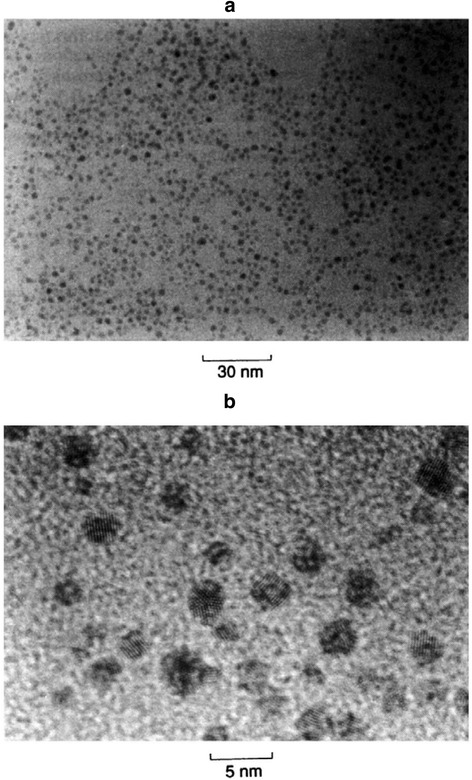
TEM pictures of the thiol derivatized gold nanoparticles at **a** low and **b** high magnification [147]. Reprinted with permission from [147]. Copyright {2010} Royal Society of Chemistry

In 1994, Brust and Schiffrin made a great contribution to the Au nanostructure synthesis by inventing a two-phase synthetic strategy. In this approach, AuCl_4_^−^ was transferred from aqueous solution to toluene using tetraoctylammonium bromide as the phase-transfer reagent and strong thiol−gold interactions were utilized to protect AuNPs with thiol ligands. Au clusters with a size range between 1 and 3 nm (Fig. 2) were obtained through the reduction reaction by sodium borohydride (NaBH_4_) in the presence of dodecanethiol [41]. As NaBH_4_ was added into the organic phase, the color of the solution turned into deep brown immediately. Several parameters including gold/thiol ratio, temperature, and reduction rate can be varied to control the size of the resulting Au nanoparticles. For example, larger thiol/gold mole ratios led to Au nanoparticles with smaller average core sizes [43]. Different ligands were utilized to form monolayer-protected gold clusters and the ratio between thiol and AuCl_4_^−^ could be adjusted in the synthesis to control the size of the AuNPs. Seed-mediated growth, developed by Jana et al. has also shown great promise for generating Au nanoparticles with controlled and monodispersed particle size [44–47]. In a typical process, high-quality seeds are required and then the cylindrical Au nanostructures are grown in multiple steps. In the seed-mediated growth approach, the yield of Au nanostructures is relatively low and high-quality seeds are necessary [48].

**Fig. 3:**
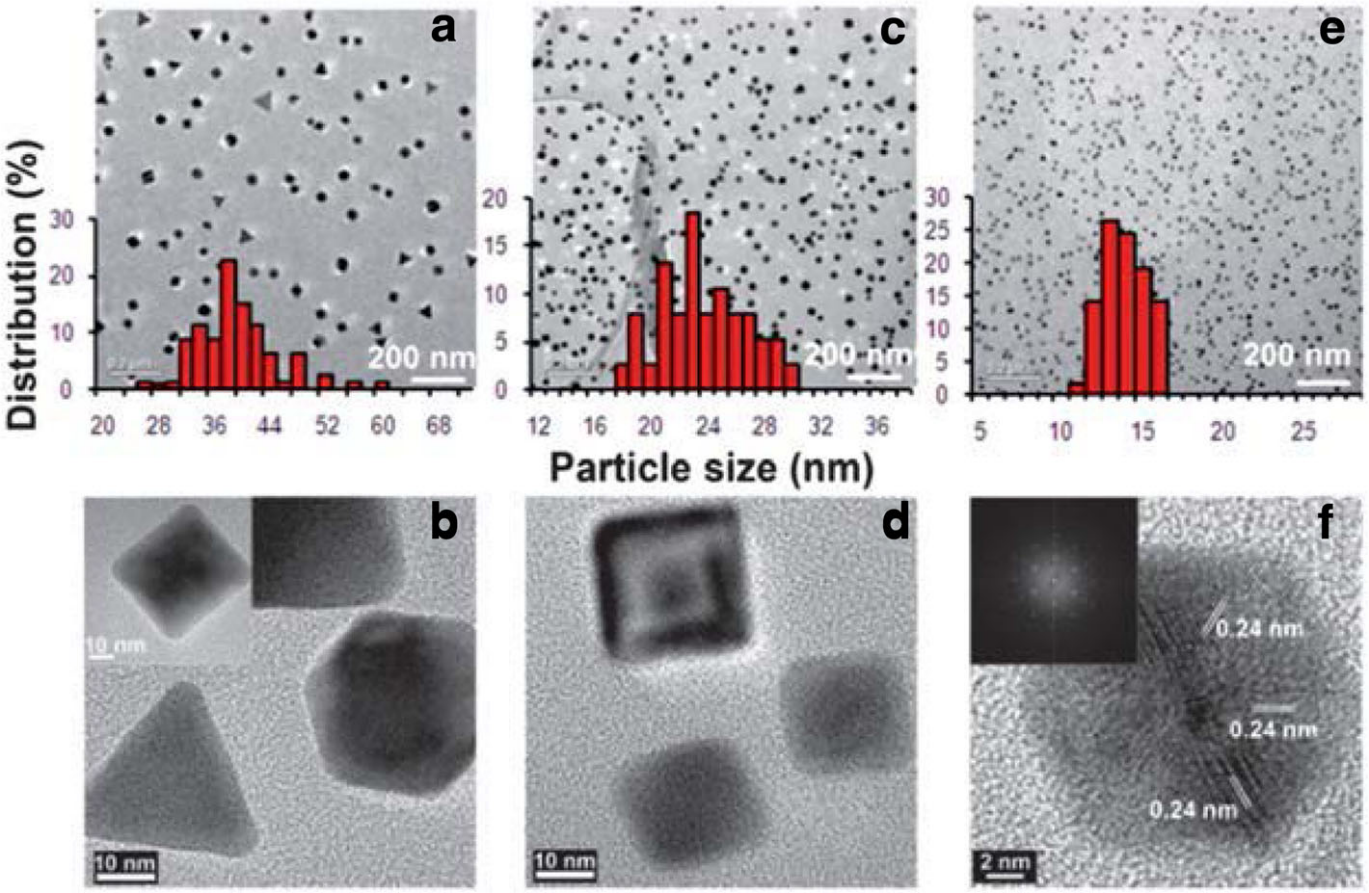
TEM images and histograms of AuNPs from AuNPs–PVP nanocomposite films with weight ratios of HAuCl4 to PVP, [HAuCl_4_/PVP] = 1:1.5 (**a**, **b**), 1:2 (**c**, **d**), and 1:4 (**e**, **f**) [88]. Reprinted with permission from ref. [67]. Copyright {2010} Royal Society of Chemistry

Various polymers have been reported for the stabilization of Au nanoparticles, which include PVP, poly(ethylene glycol) (PEG), PVA, poly(vinyl methyl ether) (PVME), chitosan, and polyethyleneimine (PEI) [49–57]. Different polymers exhibit different formation processes for Au nanostructures; for example, the reduction between gold ions and PVP may involve a solid–liquid (S–L) mechanism and the nitrogen and oxygen atom heterocyclic ring can contribute to the reducing ability of the PVP [56]. In the reduction reaction, PVP plays the roles of both a reducing agent and a steric stabilizer; therefore, by varying the concentration or ratio between PVP and Au ions, different Au nanostructures with different shapes and sizes can be achieved (as shown in Fig. 3).

**Fig. 4:**
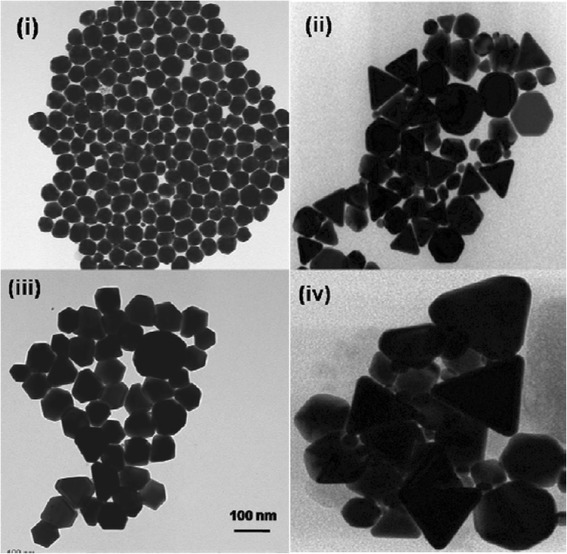
TEM images of the gold nanoparticles prepared in **i** 60, **ii** 70, **iii** 80, and **iv** 90% oleic acid [65]. Reprinted with permission from ref. [65]. Copyright {2010} American Chemical Society

In addition to the chemical synthesis strategies of Au nanostructures, several physical methods have also been used to improve the quality of the Au nanostructures, including photochemistry (UV, Near-IR), sonochemistry, radiolysis, thermolysis, and microwave irradiation [58–65]. In the microwave irradiation synthesis process, the addition of different amounts of oleic acid not only increases the growth rate but also controls the morphology of the resulting Au nanostructures as shown in Fig. 4 [65]. In addition, oleylamine could also be added as the reducing agent and the nucleated Au functions as the catalyst to initiate the reaction between oleic acid and oleylamine to form dioleamide, which plays a role as the capping agent for the as-prepared Au nanoparticles.

### Synthesis of Ag Nanostructures

Use of citrate as the reducing agent for Ag colloid synthesis in an aqueous solution has been discovered for decades. Typically, a set amount of sodium citrate solution is added into a boiling aqueous solution of AgNO_3_ and the Ag nanocrystals will be obtained after keeping the system boiling for 1 h. During the reaction, the citrate ions serve as both a reducing agent and a stabilizer and they can complex with the silver seeds, thereby influence the particle growth, leading to formation of larger clusters of silver [66]. Furthermore, by varying the pH of the solution or the concentration of citrate ions, different protonation states associated with citrate ion can be achieved, resulting in different growth mechanisms and morphologies of Ag clusters [13].

Another commonly used method for synthesizing Ag nanostructures is the polyol process, which can lead to the formation of Ag nanostructures with a wide variety of sizes and shapes [67–71]. In the polyol reduction process, the nucleation, growth process and the resultant Ag nanostructure morphology are sensitive to reaction conditions, such as temperature, reagent concentration, and presence of trace ions [13]. In a typical polyol reduction process, a Ag precursor with a capping agent is injected into a preheated polyol such as ethylene glycol; 1,2-propylene glycol; or 1,5-pentanediol, which plays a dual role as a solvent and a reducing agent [13, 66]. The exact mechanism during the polyol reduction process is still largely unknown due to its complex nature, and one of the possible reactions is as follows:

1 $$ 2\mathrm{H}\mathrm{O}\mathrm{C}{\mathrm{H}}_2\mathrm{C}{\mathrm{H}}_2\mathrm{O}\mathrm{H}\kern0.5em +\kern0.5em {\mathrm{O}}_2\to\ 2\mathrm{H}\mathrm{O}\mathrm{C}{\mathrm{H}}_2\mathrm{C}\mathrm{H}\mathrm{O} + 2{\mathrm{H}}_2\mathrm{O} $$

By using a spectroscopic method, the formation of glycolaldehyde (GA) has been confirmed and it is the intermediate product of ethylene glycol and a stronger reductant that can effectively reduce AgNO_3_ into Ag [72]. This may also explain why the polyol process is highly dependent on the reaction temperature [13]. During the polyol reaction, Ag atoms initially form small clusters and later grow into stable and bigger clusters. Finally, Ag nanostructures with different shapes and sizes will be formed after continuous growth.

Moreover, silver nitrate, in the presence of aldehyde-containing compounds (or sugar, e.g., glucose), can form Tollen’s reagent and subsequently transform into elemental Ag through the reduction reaction:

2 $$ \mathrm{RCHO} + 2\ {\left[\mathrm{Ag}{\left(\mathrm{N}{\mathrm{H}}_3\right)}_2\right]}^{+} + 2\ \mathrm{O}{\mathrm{H}}^{\hbox{-}}\to \mathrm{RCOOH} + 2\ \mathrm{A}\mathrm{g} + 4\ \mathrm{N}{\mathrm{H}}_3 + 2\ {\mathrm{H}}_2\mathrm{O} $$

This reaction is also called the silver mirror reaction, which will produce a shiny mirror coating on the inner surface of a reaction container [13]. However, no shape control can be achieved through this reaction, which limits its use in synthesizing Ag nanostructures.

Seed-mediated growth, which uses nanocrystals as seeds for further growth, has attracted a lot of attention and become another popular synthetic approach for Ag nanostructures. Basically, there are two main steps involved: the seed nucleation and the growth of the nanostructures. These two steps are essentially separate, enabling great control over the final morphology of Ag nanostructures [13, 67]. For Ag nanostructures synthesized by this method, the final shape of the nanostructure not only depends on the initial seed but is also governed by the growth rates of different crystallographic facets [13]. It has been found that the growth rates of specific facets are significantly influenced by the capping agent. For example, when used as the capping agent, citrate has been shown to bind more strongly to {111} than {100} crystal facets, tending to form nanoplates. However, for PVP, it binds more strongly to {100} than {111} crystal facets and can thereby reduce the growth rate along the [73] direction, leading to different morphology formation. These studies demonstrate that by changing the reaction conditions including capping agents and seed types,. binding strengths with different facets can be simply manipulated, leading to precise control over the morphology of the Ag nanostructures [13].

A long time ago, people found that a silver precursor (e.g., AgNO_3_) could interact with light leading to the formation of elemental silver. Therefore, in the presence of appropriate chemical species, Ag nanostructures can be formed under laser irradiation of a sample of Ag colloids [13]. Early studies found when ultrafast (femto- or nanosecond) laser pulses were applied to Ag nanostructures, these nanostructures would melt and tend to form rough spheres due to the low surface energy and thermodynamic stability of this shape [13, 74, 75]. Inspired by these early studies, later investigations revealed that light excitation could also be utilized to grow or modify nanostructures in a controllable fashion and the size and shape of the resultant nanostructures are found to be dependent on applied laser wavelength and power [76–79]. Recent studies indicate that citrate, oxygen, and light are necessary for the reaction. The mechanism behind the light-mediated synthesis is as follows: the Ag seeds that absorb/scatter light weakly reduce dioxygen and release Ag^+^ into solution; in the presence of light and Ag^+^, citrate will degrade into acetoacetate, and the resulting electrons are transferred into the Ag nanostructure, accelerating the rate of silver deposition on the surface [13, 78, 80]. By increasing irradiation light intensity, the photochemical process can be significantly enhanced which increases the photoreaction rate and the yield of Ag nanoprisms [78].

## Electrospun Polymer Nanofibers Decorated with Noble Metal Nanoparticles

Ag and Au nanostructures have proven to be versatile platforms for various application such as plasmonics, biomedical research, sensing, and catalysis [81–86]. Taking advantage of the flexibility, uniform distribution, controllable morphology, and free-standing properties of electrospun polymer nanofibers, the combination of Au or Ag nanostructures with polymer nanofibers have great potential to improve the reusability and widen the current applications. For example, it is notable that encapsulation of Ag nanoparticles into polymer fiber matrix can efficiently prevent the sulfuration on the surface of Ag nanoparticles. The addition of Au or Ag nanostructures into electrospun nanofibers can also change the nanofiber morphology. Kim and coworkers synthesized Au NP/PEO composites and found that there was a 50-nm increase of fiber diameter after addition of Au NPs to PEO (poly (ethylene oxide)) [87].

### Preparation of Electrospun Polymer Nanofibers Decorated with Ag Nanoparticles

Based on the sequence of the reduction process of Ag^+^ to Ag nanostructure, the preparation of electrospun polymer nanofibers decorated with Ag nanoparticles can be classified into two different methods. In the first method, the Ag nanostructure with different morphologies is either prepared first or the Ag precursor is reduced into Ag nanostructures inside the polymer precursor solution. If the reduction reaction is conducted in a separate solution, the as-prepared Ag nanostructures will be separated and added into the polymer precursor solution subsequently. In this way, because the Ag nanostructure reduction takes place before the electrospun nanofiber formation, it does not require a solvent that can dissolve and stabilize the Ag precursor. In addition, it is not necessary for the polymer to be able to reduce the Ag precursor and it means suitable polymers can be utilized for the composite without limitation. Furthermore, since it is easier to control the Ag nanostructure morphology in a dependent reduction process, the separate synthesis processes permit Ag nanostructure/electrospun nanofiber composites with more Ag nanostructure morphologies.

The other method to prepare electrospun polymer nanofibers decorated with Ag nanoparticles involves first dissolving the Ag precursor into the polymer precursor solution or attaching it onto the surface of the electrospun polymer nanofibers, followed by a reduction process which transforms the Ag precursor into Ag nanostructures. This method is also called the in situ growth of plasmonic nanoparticles. Generally, to perform the reduction reaction, one of the approaches is to utilize a reducing polymer or mixed polymer that contains a reducing polymer as the electrospun nanofiber precursor such as chitosan and PVP [88, 89–91]. The exact mechanism of how the PVP reduces Ag precursor into Ag nanoparticles is still not fully understood, and it has been hypothesized that the aldehyde functional groups, resulting from the oxidation of the hydroxyl end group, might reduce the metal ions in a similar way to that of Tollen’s reagent [92]. Furthermore, it is worth pointing out that the metal formation capacity is highly dependent on the molecule weight of PVP when the same mass of polymer is used [88]. Other approaches to reduce Ag^+^ in or outside the polymer nanofibers include heating, UV irradiation, microwave irradiation, or hydrogen reduction [93–97]. Leonard et al. prepared tourmaline nanoparticles/polyurethane nanofiber composite and decorated with silver nitrite on the surface [98]. After the irradiation treatment for 4 h, silver nitrate was reduced into Ag nanoparticles which exhibited a wire-like structure on the surface of the composites.

### Preparation of Electrospun Polymer Nanofibers Decorated with Au Nanoparticles

**Fig. 5:**
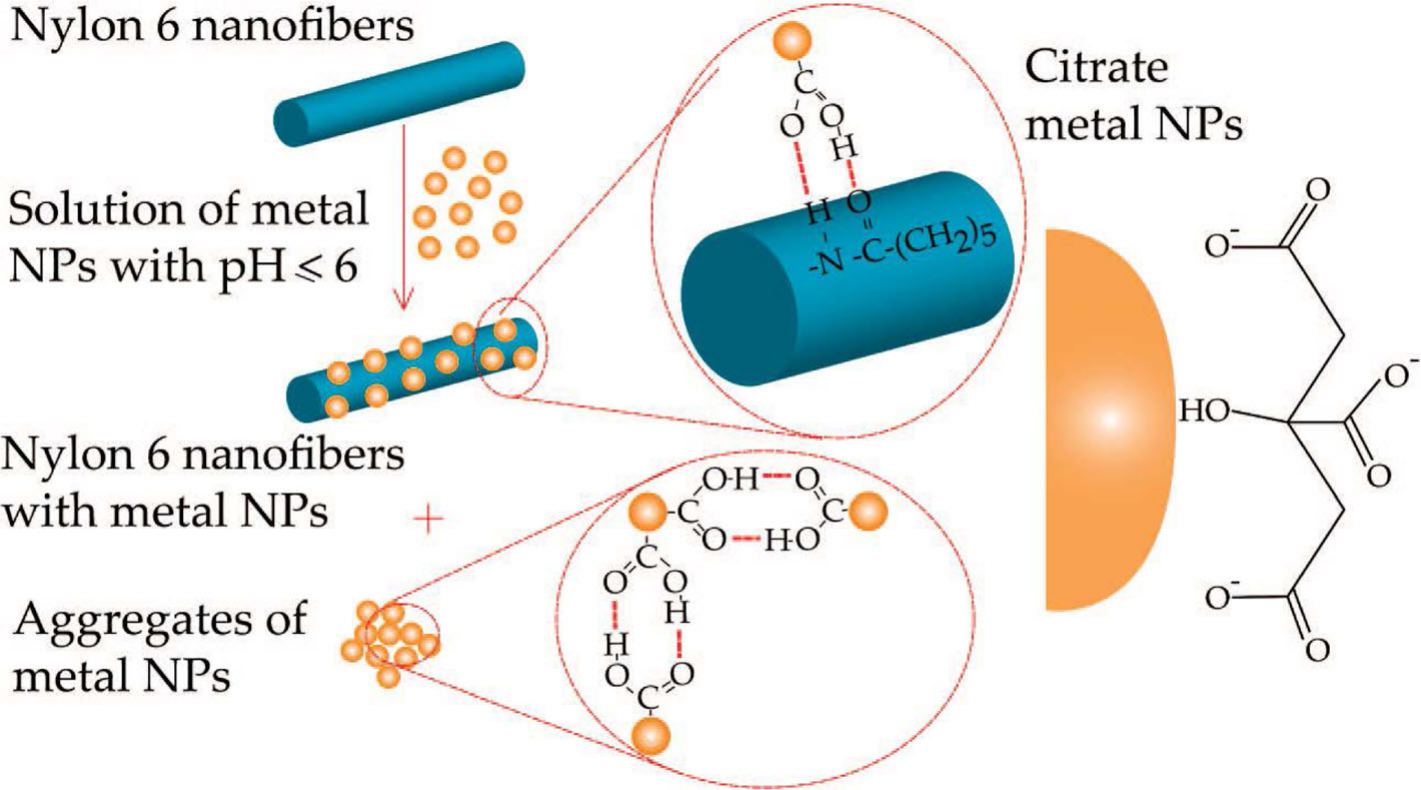
Postulated mechanism of pH-induced assembly of metal nanoparticles on the surface of nylon 6 nanofibers [103]. Reprinted with permission from ref. [103]. Copyright {2008} American Chemical Society

Similar to the strategies to encapsulate Ag nanostructures, most researchers demonstrate that electrospun polymer nanofibers decorated with Au nanoparticles could firstly be synthesized using a regular Au nanostructure method such as citrate reduction and seed-mediated approach and then disperse the as-prepared Au nanostructures into the electrospinning polymer precursor solution [73, 99–102]. For some specific applications, Au nanostructures are required to decorate on the surface of the nanofibers and Au nanostructures are found to be attracted by some specific functional groups on the polymers. By adjusting the Ag or Au solution pH, Dong et al. found that one of the three COONa groups from the surface-bound citrate on the NPs would become COOH, which could bridge the amide group on the surface of the nylon 6 fibers through two intermolecular hydrogen bonds and bond the Ag or Au NPs on the surface of the nylon 6 nanofibers as shown in Fig. 5 [103].

Some polymers contain functional groups on their backbones that can be easily modified with other materials such as 3-mercaptopropyltrimethoxysilane (MPTES) to provide stronger binding sites to attract the Au nanoparticles [99]. In addition, some polymers can be used to stabilize Au nanostructures and function as the electron donor in the reduction process of Au^3+^ to Au^0^. Pucci et al. found that under irradiation, the RCH_2_OH in the PVA with available α hydrogen atoms could be transformed into RCHO while releasing H^+^ and e^−^ [52]. Subsequently, the produced e^−^ might be trapped by the Au^3+^ to produce Au^0^ making PVA additives more efficient photo-reduction reactions [104].

## Sensing Applications of Electrospun Polymer Nanofibers Decorated with Noble Metal Nanoparticles

Some recent examples of sensing applications based on electrospun polymer nanofibers decorated with noble metal nanoparticle (e.g., Au and Ag NPs) are illustrated in Table 1. From Table 1, it is seen that the metal particle/nanofiber composites have many advantages such as simplicity, high sensitivity, and high selectivity in detecting various biological and chemical specimens. Through electrospinning, the metal particle/nanofiber composites can be easily fabricated with a high surface area, which can provide easy access for the detection molecules leading to excellent activities for SERS. Therefore, many metal particle/nanofiber composites show low limit of detection. In addition, based on the results in Table 1, it can be concluded that the density and size of metal particles have an important impact on the SERS activity/sensitivity because enhancement of Raman signals result from the presence of hot spots between/among metal particles.

**Table 1:** Examples of sensing applications of electrospun polymer nanofibers decorated with noble metal nanoparticles

Detection mode	Nanofiber materials	Au or Ag nanostructures	Limit of detection	Reference
SERS	Cellulose	Ag NPs	1 ppm thiabendazole	[118]
SERS	PVA	Au nanorods with Ag nanowires	10^−4^ M 3,3'diethylthiatricarbocyanine iodide	[117]
SERS	PVA	Au nanorods	10^−4^ M 3,3'diethylthiatricarbocyanine iodide	[100]
SERS	PVA	Ag NPs	1 μM 4-mercaptobenzoic acid (4-MBA)	[116]
SERS	PVA	Au and Ag NPs	4-MBA (2 mM) and thiophenol (1 mM)	[123]
SERS	PAA/PVA	Au NPs	10^−8^ Rhodamine 6G (R6G) and 10^−9^ 4-Aminothiophenol (4-ATP)	[115]
SERS	Poly(2-vinyl pyridine)	Au nanorods	1 mM 1,4-benzenedithiol	[73]
SERS	PVP	Ag nanowires	5 mg/mL 4,4'-bipyridine	[122]
SERS	PAN	Ag nanoparticles	10^−4^ p-Aminothiophenol	[148]
SERS	PAN	Ag NPs	10 ppb R6G	[114]
SERS	Silica	Au and Ag NPs	1 mM MBA	[121]
SERS	Chitosan	Ag NPs	1 μM R6G and 0.001 mg/mL d-glucose	[106]
SERS	PMMA	Au NPs	0.1 nM malachite green isothiocyanate	[119]
SERS	PMMA	Ag NPs	1 mM 4-MBA	[149]
Electrochemical	Bacteria cellulose	Au NPs	1 μM H_2_O_2_	[150]
Electrochemical	PAN	Ag−Pt Bimetallic NPs	0.11 μM dopamine (DA)	[151]
Electrochemical	PVA	Au NPs	0.5 μM H_2_O_2_	[99]
Electrochemical	PVA/poly(ethyleneimine)/glucose oxidase	Au NPs	0.9 μM glucose	[146]

### Electrospun Polymer Nanofibers Decorated with Noble Metal Nanoparticles for Chemical Sensing Based on SERS

Surface-enhanced Raman scattering (SERS) has emerged as one of the most promising and powerful analytical tools for probing single molecules, ions, biomolecules, and for cell studies [105–111]. Since the mid-1980s, more researchers began to focus on the exploration of promising analytical applications of SERS instead of the fundamental understanding of the phenomenon [112]. Organized Au or Ag nanostructures have attracted tremendous attention due to their signal-amplifying function as SERS substrates, which have been attributed to a local electromagnetic field enhancement induced by the metallic nanostructures. The SERS enhancement factor (ratio between the Raman signals from a given number of molecules in the presence and in the absence of the nanostructure) is closely related to the size and shape of the nanostructures that give rise to the effect [113]. Typically, the Au, Ag, or AuAg-mixed nanostructures are arranged on rigid materials as the SERS substrate and these methods are either complicated and time consuming in synthesis processes or require strict synthetic conditions.

Recently, a flexible substrate fabricated by combining electrospun nanofibers with Au, Ag, or AuAg-mixed nanostructures has become popular due to their excellent SERS performance and, compared with the rigid substrate, these flexible structures are adaptable to a rough substrate in terms of wrapping and bending [106, 114]. These metal/nanofiber composites demonstrated a 3D structure, which can provide high density of “hot spots”, which refers to the regions of highly enhanced local electromagnetic field [115]. In addition, the polymer outside the nanostructures can protect them from the surrounding environment especially for Ag nanostructures, which gives the composite long lifetime and high sensitivity [116].

**Fig. 6:**
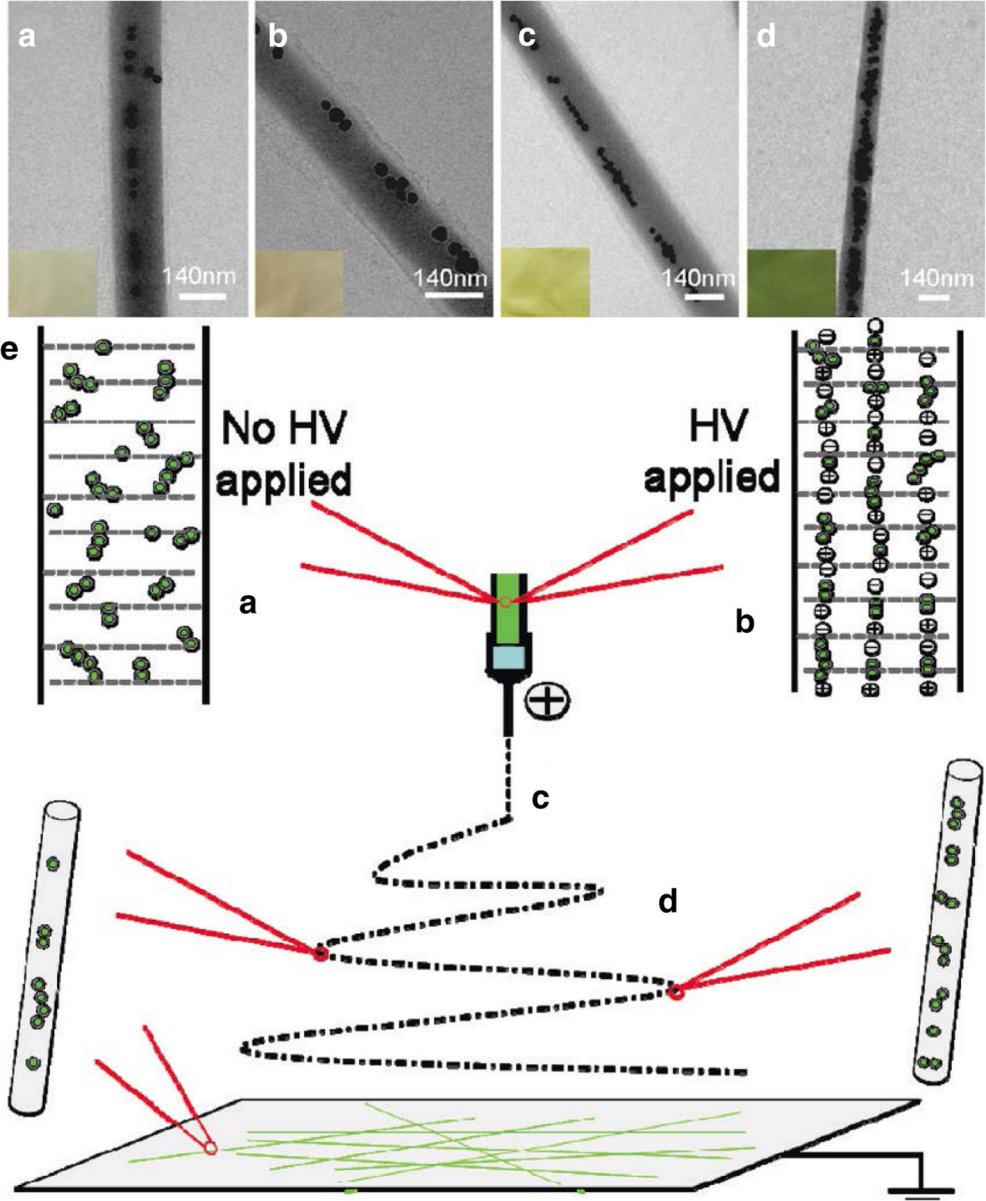
**a**–**d** Typical TEM image of Ag/PVA nanofibers with the molar ratio of PVA/Ag 530:1 (**a**), 530:2 (**b**), 530:3 (**c**), and 530:4 (**d**). The increase of the molar ratio of Ag/PVA in the Ag/PVA solution led to stronger aggregation state and a larger distribution in the sizes of the aggregated Ag NPs. **e** Schematic representation of the formation of chain-like arrays of Ag NP aggregates within PVA nanofibers [116]. Reprinted with permission from ref. [116]. Copyright {2009} American Chemical Society

Different polymers or ceramic nanofibers, such as PVA [100, 116, 117], cellulose [118], poly(methyl methacrylate) (PMMA) [119], chitosan [106], poly (acrylic acid) (PAA)/PVA [120], and silica [121] have been utilized to combine with different Ag or Au nanostructures to fabricate the flexible substrate for SERS. PVA is a nontoxic, biocompatible polymer, which has good electrospinability, and it is a popular material for electrospinning. When it is used as the supporting material for Ag nanostructures for SERS, it functions not only as the host matrix but also as an organic additive inducing the aggregation of individual Ag nanostructures [116]. In a typical process, the Au or Ag nanostructures are produced in specific morphologies first and these nanostructures are added into the polymer solution as the precursor solution. He et al. synthesized nearly monodispersed Ag NPs via a microwave-assisted method and then these as-prepared silver dimers and aggregates were mixed into a 7% aqueous PVA solution for electrospinning [116]. In order to reduce the specific surface area and the surface Gibbs free energy of individual nanofibers, Ag NPs were self-assembled inside the PVA nanofibers. The assembly of Ag dimers or aligned aggregates within PVA nanofibers was confirmed using transmission electron microscopy (TEM) and X-ray photoelectron spectroscopy (XPS) analyses. Moreover, Ag NPs tended to form a linear chain-like structure along the axial direction of fibers (Fig. 6) because when a high voltage was applied to the solution, Ag NPs became positively charged on one side and negatively charged on the other, leading to a self-alignment by electrostatic attraction in the direction of the electric field [116].

As the amount of Ag NPs increased in the PVA nanofibers, the enhancement factor did not increase accordingly, which indicated that different morphology of Ag NP aggregation had a great influence on the enhancement effects of SERS [116]. When 4-mercaptobenzoic acid (4-MBA) was used as a probing molecule to study the Raman enhancement effects, the Ag/PVA nanofibers showed excellent detection reproducibility (i.e., the average relative standard deviation values of the major Raman peak were less than 0.07). Taking advantage of the same nanoparticle alignment in the polymer nanofibers, Ag nanowires (NWs) were also synthesized and electrospun into PVA nanofibers [122]. The Ag NW/PVA nanofibers showed similar morphology and the NWs were “frozen-up” within the polymer fibers. In addition, the electrospun Ag NW/PVA nanofibers were arranged into different structures and stronger SERS intensities were obtained from the arranged samples [122]. Besides Ag nanostructures, Au nanostructures were also encapsulated into the PVA electrospun nanofibers as SERS substrates [100]. Zhang et al. used a seed-mediated surfactant-directed approach to synthesize Au nanorods (AuNRs), and these Au NRs exhibited good alignment along the axial direction of the nanofibers, which demonstrates that electrospinning is a powerful tool to assemble anisotropic nanorods on a large scale [100]. Ag and Au nanostructures can be co-assembled into the PVA nanofibers [117, 123]. Different SERS effects can be obtained by varying the Au/Ag ratio and the excitation wavelength due to the different activities of Au and Ag nanostructures under different wavelengths [124]. In spite of the different morphologies of Ag and Au nanostructures, both Au/PVA and Ag/PVA composites showed excellent SERS performance.

### Electrospun Polymer Nanofibers Decorated with Noble Metal Nanoparticles for Chemical Sensing Based on Electrochemical Techniques

Nowadays, metal nanoparticles (such as Au, Ag, Cu, and Ni) have become widely utilized in electrochemical sensing applications, which can be attributed to their rich electronic properties, high surface area, and excellent chemical stability [125, 126]. Au NPs can decrease the overpotentials of many electroanalytical reactions and maintain the reversibility of redox reactions [41, 127]. The Au NP platform can be used for detection of different kinds of analytes including small molecules such as glucose [128, 129], dopamine [130–133], bisphenol A [134], toxic chemicals and drugs such as mercury [135–138], antimony [139], and hydrogen peroxide [140]. Au NPs hold great promise as substrates for designing electrochemical biosensors, which benefit from their ability to provide a stable immobilization of biomolecules retaining their bioactivity, ease of use in chemical synthesis, narrow size distribution, and their convenient labeling of biomolecules [141–143]. Furthermore, both Ag and Au NPs have good biocompatibility and large surface area which can help adsorb biomolecules strongly and play an important role in the immobilization of biomolecules [144]. Accordingly, combining Au or Ag NPs with large-surface-area polymer nanofibers, which provide a large loading capacity for nanoparticles, can further enhance the sensitivity of the sensors [145].

Sapountzi et al. decorated the PVA/poly(ethyleneimine) (PEI)/glucose oxidase nanofibers with Au NPs to further improve the conductivity of the mat and used these composites as electrochemical biosensors [146]. However, both PVA and PEI are water soluble polymers and it may weaken the stability of the composite. Therefore, the researchers conducted a post-electrospinning cross-linking step by exposing the NFs to glutaraldehyde (GA) vapors and the morphology of the fibers was still well retained, suggesting a successful chemical cross-linking reaction induced by GA vapors [146]. The same treatment was also performed by other researchers and the cross-linked PVA nanofiber mat maintained its morphology even after being soaked in water for 15 days [99]. After obtaining the water soluble PVA nanofiber mat, 3-mercaptopropyltrimethoxysilanes (MPTES) were first modified on the surface of electrospun PVA nanofibers. Then, the modified PVA nanofiber mat was immersed into the as-prepared Au NPs aqueous solutions and Au NPs were strongly bonded onto the surface of the modified PVA nanofibers due to the strong affinity between the thiol groups and Au NPs [99]. Au NPs were homogenously decorated on the surface of the modified PVA nanofibers for different Au NP concentrations, leading to highly sensitive detection of H_2_O_2_ and the Au NPs/modified PVA also showed more advantages such as fast response, broad linear range, and low detection limit [99].

## Conclusions

Extensive research has been carried out to study the properties and applications of both Au or Ag nanostructures and electrospun nanofiber materials in recent years. Taking advantage of the flexibility, large surface area, ease of production, and surface modification of the electrospun polymer nanofibers, the combination of Au/Ag nanostructures with nanofibers makes these composites versatile platforms for various applications in optics, antibacterial coatings, photovoltaics, and chemical and biological sensors etc. The adaptable functionalization of both electrospun nanofibers and Au or Ag nanostructures can lead to unique morphologies and structures for Au or Ag nanostructure/electrospun nanofiber composites, followed by more applications with enhanced performance.

Despite the increasing number of publications using electrospun polymer nanofibers decorated with noble metal nanoparticles for sensing applications, the field is in its infancy. The rational integration of noble metal nanoparticles to nanofiber matrices to achieve desirable plasmonic properties will bring unprecedented strategies for sensor development. Further investigations are required to better understand the morphology control, formation mechanism, and applications to specific applications. It is expected that further development of this field will eventually make a wide impact on many areas of research.

## Abbreviations

GA: Glutaraldehyde
MPTES: 3-Mercaptopropyltrimethoxysilane
NWs: Nanowires
PAA: Poly (acrylic acid)
PAN: Polyacrylonitrile
PEG: Poly(ethylene glycol)
PEI: Polyethyleneimine
PEO: Poly (ethylene oxide)
PVA: Polyvinyl alcohol
PVdF: Polyvinylidene fluoride
PVME: Poly(vinyl methyl ether)
PVP: Polyvinylpropelene
SERS: Surface-enhanced Raman scattering
TEM: Transmission electron microscopy
XPS: X-ray photoelectron spectroscopy
